# Formulation and Evaluation of Plumbagin-Loaded Niosomes for an Antidiabetic Study: Optimization and In Vitro Evaluation

**DOI:** 10.3390/ph16081169

**Published:** 2023-08-17

**Authors:** Rama Tyagi, Ayesha Waheed, Neeraj Kumar, Abdul Ahad, Yousef A. Bin Jardan, Mohd. Mujeeb, Ashok Kumar, Tanveer Naved, Swati Madan

**Affiliations:** 1 Amity Institute of Pharmacy, Amity University, Noida 201303, Uttar Pradesh, India; 2 Department of Pharmaceutics, School of Pharmaceutical Education and Research, Jamia Hamdard, M. B. Road, New Delhi 110062, India; 3 Department of Pharmacognosy & Phytochemistry, School of Pharmaceutical Education and Research, Jamia Hamdard, M. B. Road, New Delhi 110062, India; 4 Department of Pharmaceutics, College of Pharmacy, King Saud University, Riyadh 11451, Saudi Arabia; 5 Department of Internal Medicine, University of Kansas Medical Centre, Kansas City, KS 66160, USA

**Keywords:** quality by design, plumbagin, diabetes, in vitro, niosomes

## Abstract

Diabetes treatment requires focused administration with quality systemic circulation to determine the optimal therapeutic window. Intestinal distribution through oral administration with nanoformulation provides several benefits. Therefore, the purpose of this study is to create plumbagin enclosed within niosomes using the quality by design (QbD) strategy for efficient penetration and increased bioavailability. The formulation and optimization of plumbagin-loaded niosomes (P-Ns-Opt) involved the use of a Box–Behnken Design. The particle size (PDI) and entrapment efficiency of the optimized P-Ns-Opt were 133.6 nm, 0.150, and 75.6%, respectively. TEM, DSC, and FTIR were used to analyze the morphology and compatibility of the optimized P-Ns-Opt. Studies conducted in vitro revealed a controlled release system. P-Ns-Opt’s antioxidant activity, α-amylase, and α-glucosidase were evaluated, and the results revealed a dose-dependent efficacy with 60.68 ± 0.02%,90.69 ± 2.9%, and 88.43 ± 0.89%, respectively. In summary, the created P-Ns-Opt demonstrate remarkable potential for antidiabetic activity by inhibiting oxygen radicals, α-amylase, and α-glucosidase enzymes and are, therefore, a promising drug delivery nanocarrier in the management and treatment of diabetes.

## 1. Introduction

1,4-naphthoquinones are a class of phytoconstituents that have been extracted from natural resources over the past few decades, and these compounds have a wide range of biological attributes and regulate a number of pharmacological functions, making them promising new targets for various illnesses [1]. A naturally occurring naphthoquinone called plumbagin (5-hydroxy-2-methyl-1,4-napthoquinone, PLB) has been identified in the roots of the traditional medicine plant *Plumbago zeylanica* L. Plumbagin has been shown to have a variety of biological effects, including anti-inflammatory, anti-cancer, antioxidant, antibacterial, antifungal, anti-atherosclerosis, and analgesic actions. It is a pro-oxidant and a vitamin K3 analog [2]. Plumbagin is known to inhibit diabetes by enhancing GLUT4 translocation and glucose homeostasis [3]. According to research, plumbagin exhibits restricted biopharmaceutical qualities, including high lipophilicity, insolubility in water, a short biological half-life, and a low melting point, which result in it having bioavailability of only 39% upon oral administration [4]. Using an alternative and correct dosage form, plumbagin can be utilized to deal with issues related to its biopharmaceutical properties. Switching from conventional form to nanoformulation-loaded natural compounds can tackle the challenges caused by solubility, penetration, toxicity, bioavailability, etc.

Niosomes are vesicular systems comprising nonionic surfactants, and they have a unique structure that enables the integration and distribution of hydrophobic and hydrophilic medicinal molecules, respectively. Niosomes are also osmotically responsive, nontoxic, immune suppressive, biocompatible, and biodegradable. Niosomes have been studied as a potential drug carrier system with a number of administration methods, including oral, parenteral, dermal/transdermal, ophthalmic, and pulmonary [5]. The findings revealed that encapsulating curcumin in niosomal nanoparticles would enhance anti-tumor results of curcumin on glioblastoma [6].According to an in vitro study, utilizing niosomes to encapsulate Law can dramatically boost the formulation’s anticancer activity in the MCF-7 cell line. Niosomes are a potentially useful delivery mechanism for phytochemical substances, since they have low solubility in bodily fluids [7]. Various niosomes have been synthesized to overcome the limitations of phytoconstituents [8].

The investigation was designed to encapsulate the plumbagin into niosomes by optimizing three independent variables: cholesterol (X1), span 20 (X2), and sonication time (X3). Vesicle size (Y1), entrapment effectiveness (Y2), and PDI (Y3) were used as dependent variables to optimize the niosomes. The antioxidant activity (DPPH assay) and invitro diabetic assay of the optimized formulation were also assessed.

## 2. Results and Discussion

### 2.1. Independent Variables Impact on Vesicle Size 

The study revealed that cholesterol has a optimistic impact, while Span 20 and sonication time have a negative impact, on vesicle size, as shown in the equation below:

Y1 = +86.49583 + 1.26000X1 − 1.74125X2 + 5.69792X3 − 0.001500X1 × X2 − 0.010000X1 × X3 + 0.006250X2 × X3 + 0.006635X12 + 0.018292X22 − 1.00521X32

**Figure 1:**
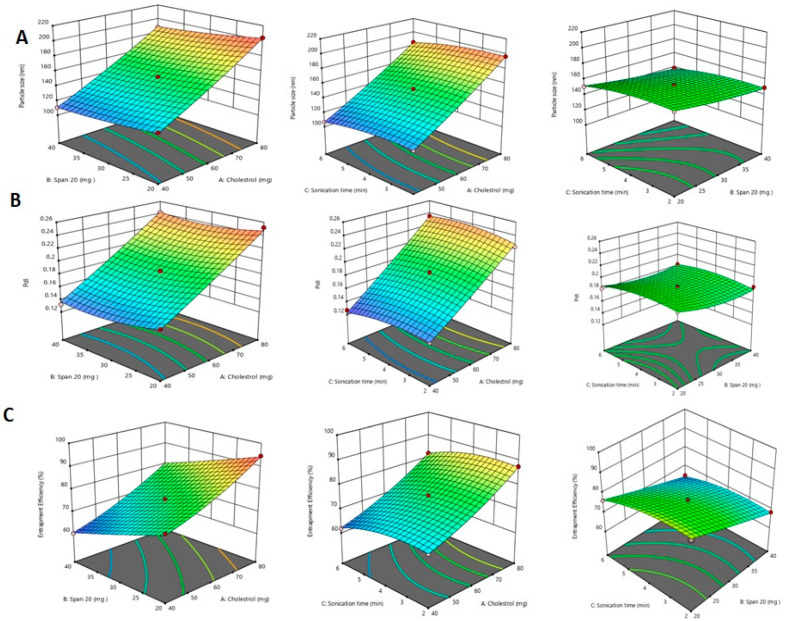
A 3D response surface plot showing the effect of independent variables on (**A**) vesicle size, (**B**) PDI, and (**C**) %EE.

The P-Ns formulation exhibited the lowest vesicles size of 106.4 nm (P-Ns-11), while the highest vesicles size of 204.4 nm was recorded for the P-Ns-15 formulation. The P-Ns-11 formulation presented minimum vesicles of 106.4 nm in size and composed of cholesterol (40 mg), Span 20 (30 mg), and sonication time (6.0 min), and the P-Ns-15 formulation presented maximum vesicles of 204.4 nm in size and composed of cholesterol (80 mg), Span 20 (20 mg), and sonication time (4 min) (Table 1). Our findings revealed that P-Ns’ vesicles size increases as the concentration of cholesterol increases (Figure 1). It was noted that the formulations that contained 80 mg of cholesterol showed higher vesicles sizes, such as P-Ns-2 (196.8 nm), P-Ns-3 (185.1 nm), and P-Ns-4 (187.9 nm), while P-Ns-1, P-Ns-5, P-Ns-7, and P-Ns-11 formulations, which included 40 mg of cholesterol, produced vesicles with lower sizes of 110.3 nm, 133.6 nm, 116.5 nm, and 106.4 nm, respectively. Changes in vesicle size that augmented the cholesterol concentration could be attributed to various factors, such as strength, elasticity, membrane rigidity, and solubility [9]. It was noted that smaller vesicles might be produced by increasing the concentration of the surfactant (Span 20) [10]. As the Span 20 concentration increases from 20 to 40 mg, the sizes of vesicles decrease from 133.6 to 110.3 nm, 162.3 to 138.7 nm, and 150.9 to 106.4 nm, respectively, as can be observed in the P-Ns-5 and P-Ns-1, P-Ns-6 and P-Ns-10, and P-Ns-13 and P-Ns-11 formulations. Additionally, it was found that a longer sonication period results in smaller vesicles [11]. The P-Ns-2 formulation prepared with less sonication (2 min) displayed bigger vesicles size than the P-Ns-8 and P-Ns-10 (4 and 6 min) formulations (Figure 1A). 

### 2.2. Independent Variables Impact on PDI 

The results of the trial suggest that the independent variables cholesterol and Span 20 have positive effects on PDI, as shown in the equation below.

Y2 = +0.165583 + 0.001206X1 − 0.005938X2 + 0.001542X3 + 0.000010X1 × X2 +  0.000119X1 × X3 + 0.000113X2 × X3 + 4.79167 × 10^−6^X12 + 0.000074X22 − 0.001583X32

For the P-Ns-15 formulation, the maximum PDI was reported to be 0.252, while the minimum PDI was observed to be 0.127 (Figure 1B, Table 1). When the amount of cholesterol was increased from 40 to 80 mg, it was found that the PDI increased immediately from 0.132 (P-Ns-1) to 0.252 (P-Ns-15), while with Span 20, vesicle size decreased upon increasing the quantity from 20 to 40 mg (P-Ns-15 and P-Ns-10), and the sonication time was shown decrease in PDI (0.193, 0.150 and 0.127) as the duration of sonication increased from 2 to 4 and 6 min in formulations P-Ns-6, P-Ns-5, and P-Ns-11, respectively, maintaining the same values for the Span 20 (X2) and sonication time (X3). This finding indicates that Span 20 (X2) has a significant impact on PDI. In comparison, the P-Ns-1, P-Ns-8, and P-Ns-6 formulations, which had the highest to lowest concentrations of Span 20, exhibited lower to greater PDI. 

### 2.3. Independent Variables Impact on Entrapment Efficiency%

In the current investigation, cholesterol had a favorable effect, while Span 20 had a detrimental impact on the effectiveness of entrapment of P-Ns vesicles, as mentioned in the equation below.

Y3 = +87.37917 − 0.082500X1 − 1.05625X2 + 2.50208X3 + 0.000750X1 × X2 + 0.001250X1 × X3 + 0.038750X2 × X3 + 0.004552X12 + 0.002458X22 − 0.638542X32

P-Ns-15 and P-Ns-1 had the highest and lowest entrapment efficiencies, which were found to be 94.6% and 60.1%, respectively (Table 1). In contrast to Span 20, cholesterol (X1) had a favorable impact on %EE, while Span 20 (X2) had a negative impact (Figure 1C). It was shown that, as demonstrated in the P-Ns-11 and P-Ns-7, P-Ns-9 and P-Ns-6, and P-Ns-3 and P-Ns-15 formulations, an increase in the content of cholesterol (40 mg–80 mg) increased the %EE from 61.8 to 67.6%, 75.4 to 83.1%, and 81.9 to 94.6%, respectively. The EE% decreased in the P-Ns-15 and P-Ns-6, P-Ns-3 and P-Ns-8, and P-Ns-1 and P-Ns-11 formulations from 94.6 to 83.1%, 81.9 to 75.2%, and 61.8 to 60.1% when the concentration of Span 20 was increased from 20 to 40 mg. The %EE steadily dropped as the sonication period increased. Ultra-sonication of formulations not only aided in shrinking vesicles, but also had an impact on drug entrapment by breaking vesicles, which caused drug(s) to leak from vesicles and reduced drug entrapment. As seen in P-Ns-2 and P-Ns-6, P-Ns-4 and P-Ns-8, and P-Ns-13 and P-Ns-10 formulations, increasing the duration of the sonication time (2 min to 6 min) decreased the %EE from 87.5 to 83.1%, 79.2 to 75.2%, and 76.3 to 64.7%.

### 2.4. Point Prediction

For the purpose of choosing an optimum formulation and performing additional characterization, the point prediction approach was utilized. The optimized P-Ns-Opt formulation had cholesterol (40 mg), Span 20 (20 mg), and 4 min of sonication. The identification of the actual and predicted values of all of the dependent variables was also examined. PDI was 0.150, entrapment efficiency was 75.6%, and the actual vesicle size was 133.6 nm. According to the expected parameters, the vesicle was 152.56 nm in size, having a PDI of 0.150 and entrapment effectiveness of 75.43%.

### 2.5. Vesicle Dimensions and PDI

**Figure 2:**
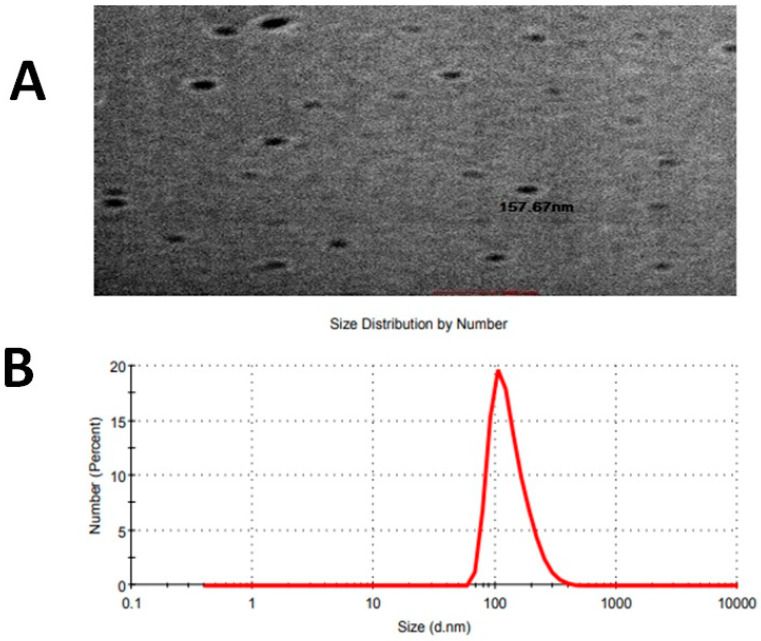
(**A**) TEM image of optimized formulation P-Ns-Opt and (**B**) vesicle size.

The optimized plumbagin-loaded niosomes had particles with a size of 133.6 nm, as determined using dynamic light scattering and a nano-zeta sizer. An extraordinarily low PDI of 0.150 for the particle size distribution was discovered (Figure 2A).

### 2.6. Morphological Study

The morphology and existence of the P-Ns-Opt formulation were verified using the TEM micrograph. The micrograph of the optimized P-Ns-Opt formulation (Figure 2B) showed a homogeneous distribution of sizes and spherical-shaped sealed formations.

### 2.7. Thermal Analysis

**Figure 3:**
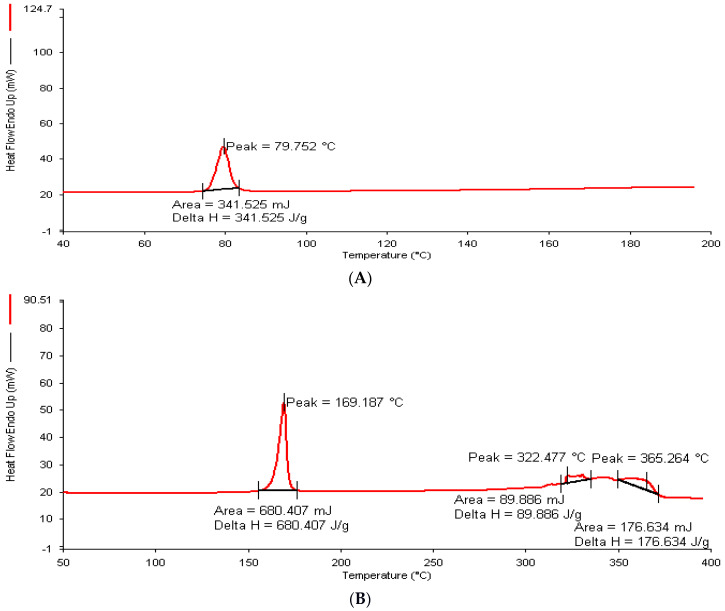
DSC image of (**A**) plumbagin and (**B**) P-Ns-Opt.

According to the supplier’s certificate of analysis, plumbagin has a melting point between 76 and 78 °C. A strong endothermic peak at 79.752 °C was seen during the DSC analysis of pure plumbagin, confirming its purity (Figure 3A). The DSC of the lyophilized P-Ns-Opt (Figure 3B) showed a peak at 169.187 °C, which belonged to mannitol. Additionally, it was noted that there were no additional peaks of available free drug in the thermogram, suggesting that there had been no precipitation and the drug had been entirely enclosed in the vesicles. As a result, it can be inferred that cholesterol and Span 20 interact or combine with one another, and this finding verifies the production of plumbagin niosomes and the absence of the anticipated plumbagin leaking.

### 2.8. FTIR Analysis

**Figure 4:**
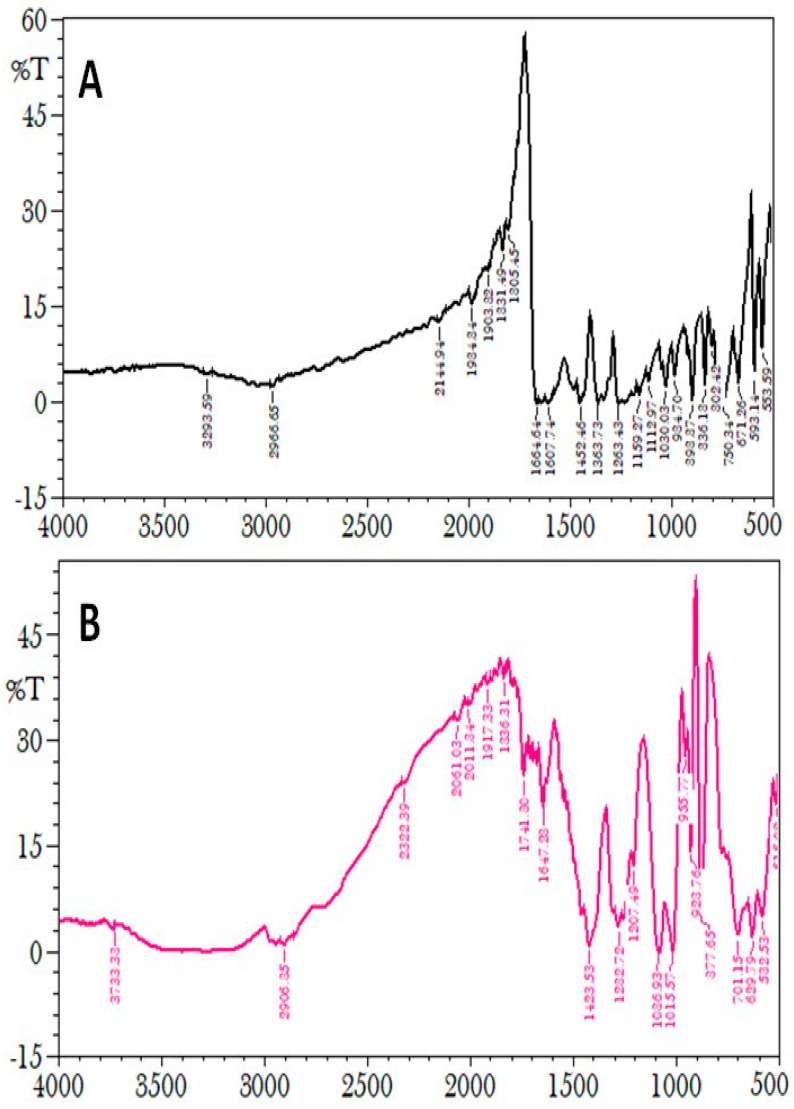
FT-IR spectra of (**A**) plumbagin and (**B**) P-Ns-Opt.

To evaluate the compatibility with excipients, the FTIR was used to evaluate the plumbagin and P-Ns-Opt formulations (Figure 4). Peaks at 3293.59, 2966.65, 2144.94, 1984.84, 1903.32, 1805.45, 1664.64, 1452.46, 1263.43, 1112.97, 1030.03, 934.70, and 898.87 cm^−1^ were visible in the characteristic spectra of the plumbagin sample. Peaks at 3733.33, 2906.25, 2322.39, 2011.34, 1741.90, 1647.23, 1423.53, 1232.72, 1036.93, 1015.57, 923.76, and 877.65 cm^−1^ were shown by the P-Ns-Opt. The FTIR spectra showed the standard sample peak, which was also present in the spectra peaks of P-Ns-Opt, demonstrating the unaltered attachment of the plumbagin molecule derived from the optimized formulation to the excipients.

### 2.9. In vitro Drug Release

**Figure 5:**
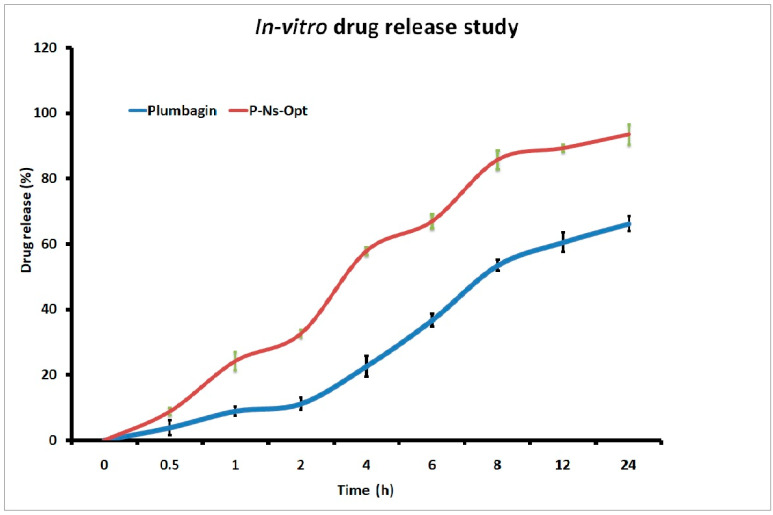
Comparative drug release of plumbagin and plumbagin-Ns-Opt.

P-Ns-Opt and plumbagin dispersion were compared on the basis of the drug release behavior (Figure 5). After 6 h of the trial, 36.6% of the cumulative release of plumbagin from the plumbagin suspension was seen to be rapidly released. After 24 h, a release rate of 66.17% was seen. While P-Ns-Opt demonstrated prolonged drug release, it also displayed an initial rapid release that might have been brought on by the unentrapped drug. Later, P-Ns-Opt displayed a plumbagin release rate of 88.19% at 12 h and a release rate of 93.04% at 24 h. The plumbagin was first released in bursts over a few hours, as shown in Figure 5, and the release was then continued for up to 24 h. By replenishing the receiver medium, the new buffer was employed to preserve the sink condition; however, this cannot entirely replicate the sink condition. As a result, it might not be able to completely remove pharmaceuticals from the matrix.

Several drug release kinetic models, including the Korsmeyer–Peppas, Higuchi, zero-order, and first-order models, were employed to assess the drug release data. The Korsmeyer–Peppas model had the greatest R2 value (0.971), the Higuchi model had R2 = 0.891, the first-order model had R2 = 0.488, and the zero-order model had the lowest R2 value (0.666). As a result, the release of plumbagin from P-NS-Opt showed the best-fitting model to be the Korsmeyer–Peppas model.

### 2.10. Antioxidant Study

**Figure 6:**
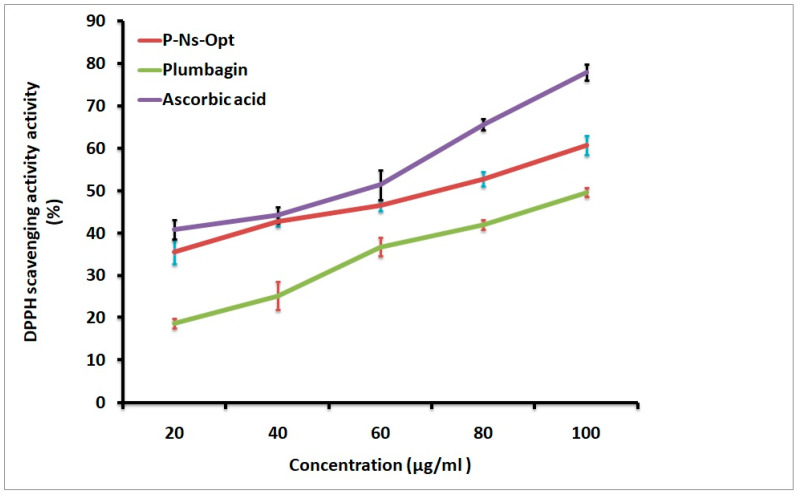
DPPH scavenging activity of Ascorbic acid, P-Ns-Opt, and plumbagin.

To inhibit, oppose, or delay a reaction, antioxidants can bind with free oxygen radicals in a chemically synthesized or natural way. In this study, the antioxidant capabilities of the ascorbic acid, P-Ns-Opt, and plumbagin were evaluated using the DPPH method (Figure 6). Ascorbic acid, plumbagin, and P-Ns-Opt each had antioxidant activities of 77.86 ± 0.03%, 49.59 ± 0.06, and 60.68 ± 0.02%, respectively. Thus, it is clear that the produced ascorbic acid and P-Ns-Opt have greater potential than plumbagin to treat a variety of disorders linked to oxidative stress.

### 2.11. Inhibitory Assay of α-Amylase and α-Glucosidase Assay

**Figure 7:**
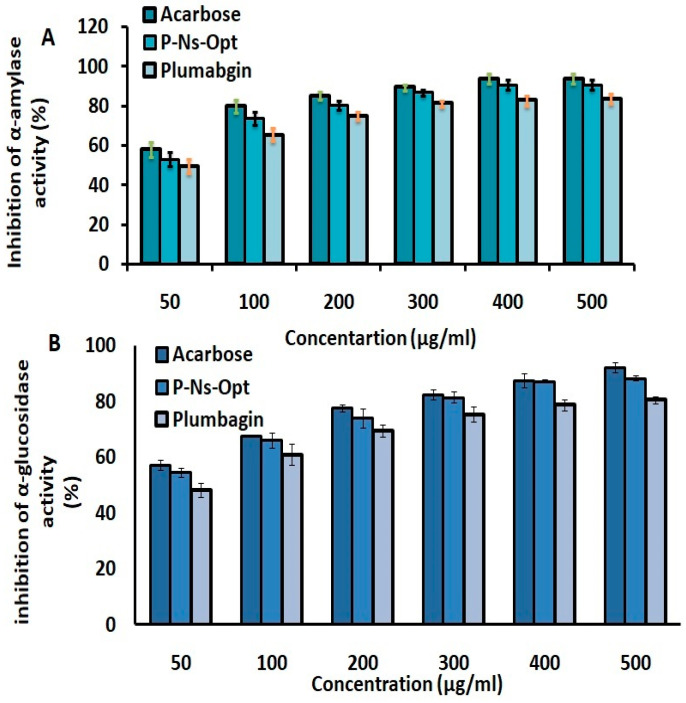
Inhibition of (**A**) α-amylase and (**B**) α-glucosidase activity of plumbagin and P-Ns-Opt.

The primary enzymes that assist in breaking apart glucose and speed up the way in which it is absorbed by the gastrointestinal tract are α-amylase and α-glucosidase, which result in higher level of sugar in blood and the suppression of the enzymes responsible for the digestion of carbohydrates, leading to the lowering of blood sugar levels. Figure 7 depicts the inhibitory effects of plumbagin, P-Ns-Opt, and acarbose. Standard (acarbose) suppressed α-amylase at the maximum level, followed by P-Ns-Opt and plumbagin at 93.71 ± 2.6%, 90.69 ± 2.9%, and 83.64 ± 3.5%, respectively. The results of the α-glucosidase inhibition assay showed that acarbose exhibited greater levels of inhibition effectiveness (93.16 ± 1.7%) than P-Ns-Opt (88.43 ± 0.89%) and plumbagin (81.07 ± 1.2%).

### 2.12. Stability Study

The P-Ns-Opt formulation’s stability studies were conducted to assess its durability during storage (8 weeks) and any medication degradation/loss stemming from niosomes (Table 2). At the conclusion of the first month, the EE% and vesicle size of the P-Ns-Opt formulation maintained at 4 °C/60 ± 5% RH were assessed. The measurements of EE% and vesicle size were 69.93 ± 2.1% and 166.56 ± 6.3 nm, respectively. The remaining medication was once more assessed after 8 weeks using the same technique as that used to assess the entrapment effectiveness and vesicle size. The findings indicated that the vesicles were stable under refrigeration and the EE% were 63.71 ± 2.7% and 180.96 ± 1.8 nm, respectively.

## 3. Materials and Methods

### 3.1. Materials

Span 20, cholesterol, disodium hydrogen phosphate, and potassium dihydrogen phosphate were procured from SD Fine chemicals, Mumbai, India. Plumbagin was purchased from Sigma-Aldrich. Methanol, ethanol, sulfuric acid, and HCl (LR grade and AR grade) were purchased from Merck, Mumbai, India.

### 3.2. Methods

#### 3.2.1. Encapsulation of Niosomes Loaded with Plumbagin

The solvent evaporation method approach was used to manufacture P-Ns-Opt. The recipe used cholesterol, Span 20, and plumbagin as components. Each ingredient was measured out in the required amounts, before being dissolved in ethanol. For thorough mixing, samples underwent a 10-minutevortexing process. Separately for one hour at room temperature, a magnetic stirrer was used to continuously mix the surfactant solution at 800 rpm while the ethanolic lipid mixture was introduced using a syringe. The obtained dispersions were probe sonicated in the presence of ice after the ethanol had been completely removed in order to produce nano-sized niosomes (NNs). The niosomes were assessed for additional criteria.

#### 3.2.2. Optimization of Plumbagin Niosomes Formulation using Response Surface Methodology (RSM)

The three factors found at three levels were applied to enable the optimization of P-NNs using Design–Expert software (Version 12, Stat-Ease, MN, USA). To ascertain their impacts on the particle size (Y1), PDI (Y2) and entrapment efficiency (Y3) of niosomes, the various process parameters—cholesterol concentration (X1), Span 20 (X2), and sonication time (X3)—were thoroughly studied. To obtain the ideal composition, independent variables were mentioned at low (−), medium (0), and high (+) concentrations (Table 3). To evaluate the impact of independent variables, we included 15 distinct composition formulation runs with three center points. Polynomial equations and response surface plots were used to assess the impacts of independent variables. The polynomial equation provided numerous models, including the quadratic and linear impacts of independent factors on dependent variables. The quadratic model was the best of all the models because the variables utilized showed both individual and combined impacts on the dependent variables.

#### 3.2.3. Characterization

##### Vesicles Size and Poly Dispersity Index (PdI)

The vesicle size and PDI of P-Ns-Opt were measured using a Malvern zeta sizer (Zeta sizer, Malvern Instruments Ltd., Malvern, UK). Using Milli-Q water, analysis was performed on samples that had been diluted up to 50 times while keeping the system’s temperature at 25 °C and the scattering angle at 90°.

##### Assessment of Entrapment Efficiency

Utilizing the ultra-centrifugation method, the % entrapment efficiency was evaluated [12]. The centrifuge (Remi cooling centrifuge, Mumbai) was used to centrifuge a plumbagin-loaded niosome sample at a specified concentration for one hour at 4 °C. After appropriate dilution, the obtained supernatant was examined to determine the presence of free drugs, and quantification was carried out using the formula below.

Entrapment Efficiency = A1 − A2/A1 × 100,

where A1 is the plumbagin concentration utilized in the loaded niosomes, and A2 is the plumbagin concentration assumed to be present in the supernatant.

##### Thermal Analysis

DSC (Perkin Elmer, Pyris 6 DSC, Shelton, CT, USA) was carried out for plumbagin and lyophilized optimized P-Ns-Opt to validate the entrapment and type of drug [13]. A tiny amount of the sample was placed in an enclosed in an aluminum pan. The instrument’s temperature was set in arrange 40 to 400 °C. The rate of nitrogen flow was regulated at 60 mL/min while the heating rate increased by 10 °C/min. Mannitol was added as a cryoprotectant at a concentration of 5%, before the formulation was freeze-dried to enable lyophilization.

##### FTIR Analysis

The spectra of the prepared plumbagin niosomes and the individual plumbagin were obtained using an FTIR spectrophotometer (Perkin Elmer-spectrum RX-I, Lamba, Shelton, CT, USA) based on the KBr disc method under the influence of a hydraulic press employing 600 kg/cm^2^ pressure. FTIR was utilized to examine sample structural characteristics, drug-ingredient interactions, and compatibility [14].

##### Transmission Electron Microscopy

The morphology of the P-Ns-Opt sample was investigated via transmission electron microscopy (JEOL; 120CX Microscope, Tokyo, Japan) [15].The dispersion was applied to a copper grid with carbon coating and allowed to attach to the carbon substrate for 1 min. Further, niosomes were stained with a drop of 1% phosphotungstic acid and placed on the grid, dried, scanned, and photographed, before being stored. 

##### In Vitro Drug Release

The Franz diffusion cell was used to carry out the drug release investigation, and a (pre-treated) dialysis membrane was used. The donor and receiver chambers were then positioned on either side of the active membrane. The recipient chamber contained phosphate-buffered saline (PBS) with a pH of 5.5, was maintained at room temperature, and swirled at 100 rpm for 24 h [16]. P-Ns-Opt and plumbagin suspension (1 mg/mL) were placed in the donor compartments. At pre-determined intervals of 1, 2, 4, 6, 8, 12, and 24 h, 1-millilitersamples were taken. To equalize the sink conditions, fresh release medium was again simultaneously put in the receiver compartment. Using UV spectroscopy and the proper dilutions, the removed sample was further evaluated to determine drug presence.

%CDR = Conc. × DF × Vol. of release media/Amount of drug added × 100

where DF is the dilution factor, and Conc. is the drug concentration achieved at a certain time interval. Time vs. cumulative drug release % was shown on the X and Y axes of a release profile graph.

Different kinetic models, such as the Korsmeyer–Peppas, Higuchi, zero-order, and first-order models were used to study the drug release data derived from the P-Ns-Opt. The model with a value of R^2^ nearer to 1 was selected as the model best suited to drug release.

##### DPPH Assay

The approach outlined, followed by with a few minor modifications, was used to estimate the DPPH’s radical scavenging capacities [17]. Various dilutions of plumbagin and P-Ns-Opt sample were combined with DPPH solution and left at 37 ± 0.5 °C for 1 h (the time needed for reactions to maintain a plateau). To prepare a blank solution, methanol and DPPH were added to the control solution, sample, and DPPH solution. Ascorbic acid was used as a standard for the study. Then, its absorbance was evaluated at 517 nm. The DPPH antioxidant action was determined as a % of DPPH inhibition.

##### Inhibition of α-Amylase Activity

The starch was utilized as the substrate to analyze the inhibition of α-amylase activity using a protocol that was slightly modified [18]. Various concentrations of P and P-Ns-Opt (50 to 250 µg/mL) were prepared, and 500 µL of sodium phosphate buffer (0.02 M) with sodium chloride (6 mM) was added separately, before being stored at room temperature for 20 min. Test tubes were filled with each sample and the amylase solution, which were then incubated for 10 min at 25 °C. Following incubation, starch solution was added and incubated at 25 °C for an additional 10 min. Dinitrosalicylic acid (DNSA) color reagent was added to the reaction to stop it and further incubated via a boiling water bath at 100 °C for five minutes. After allowing the reaction mixture to reach room temperature, 3 mL of double-distilled water was added.

The absorbance was then measured at 540 nm via a microplate reader set to 25 °C using a 200-microliter aliquot of the reaction mixture. Each well had a blank reading (the addition of buffer to replace the enzyme) removed. The maltose equivalents emitted from starch at 540 nm were used to measure the enzyme activity. The same methodology was utilized to assess an artificial α-amylase inhibitor (acarbose), which served as a standard. The α-amylase inhibition percentage was calculated using the formula given below:

Percentage of inhibition = [(Ac − As)/Ac] × 100

where Ac = absorbance of the controls, and As = absorbance of the sample.

##### Inhibition of α-Glucosidase Activity

50 µL of plumbagin and P-Ns-Opt were made at various concentrations and then stored for 20 min at room temperature with 10 mL of α-glucosidase (maltase) and 125 mL of 0.1-molarity phosphate buffer (pH 6.8) [19]. Next, 20 µL of 1-molarity 4-Nitrophenyl-D-glucopyranoside was mixed to begin the reaction, which was then incubated for 30 min. The reaction was stopped by adding 50 µL of 0.1-newton Na2CO3. In order to determine the optical density, a spectrophotometer was used at 405 nm. Acarbose served as a standard. All tests were conducted in triplicate.

The inhibition percentage of P-Ns-Opt was estimated using the following formula:

Percentage of inhibition = [(Ac − As)/Ac] × 100

where Ac = absorbance of the controls, and As = absorbance of the sample.

##### Stability Study

The stability of the optimized P-Ns-Opt was evaluated while the formulation samples were kept at 25 °C and 4 °C for an 8-week period [20]. Samples were obtained at 0, 4, and 8 weeks to determine particle size and EE%, which were used as the stability parameters.

## 4. Conclusions

Plumbagin has the capacity to heal wounds, as well as having anti-diabetic, anti-inflammatory, anti-cancer, and immunosuppressive effects. Plumbagin has penetration-related issues due to the size of vesicles, solubility, and bioavailability. Such issues may be mitigated by developing nano-formulations, boosting bioavailability, including therapeutic activity, etc. 

Niosomes are one drug delivery system which has the efficiency to carry the drug through various routes, like ophthalmic, nasal, parenteral, transdermal, and oral routes. A niosome is known as a “drug depot” because it has the potential to transfer the drug in a controlled or sustained manner to the targeted site. The improved version of a loaded drugs is enclosed in niosomes by enhancing solubility and biocompatibility. Niosomes have hydrophilic, amphiphilic, and lipophilic properties. So, diverse drugs with broad levels of solubility can be accommodated via the structure of a niosome. This fact is the reason that drugs loaded in niosomes can pass through the stomach without being degraded by the acid present in the stomach. Another problem with conventional drugs involves penetration through the intestine and reaching the bloodstream. Niosomes have the capability to enhance the bioavailability of loaded drugs and promote their efficiency. Niosomes are composed of nonionic surfactants and cholesterol. Cholesterol is the carrier of the lipophilic and amphiphilic drugs. Cholesterol prepares the bilayer system, which is rigid, helps to maintain stability, and results in less leaky and larger-sized niosomes. Meanwhile, the surfactant’s HLB value shows an effect on the vesicle size. The higher the HLB value, the higher the surface energy, which will lead to a large vesicle size (higher hydrophilicity due to the higher uptake of water). 

In this investigation, we have formulated P-Ns-Opt that improved in vitro performance related to diabetes. The optimized formulation generated vesicles with diameters of 133.6 nm, a PDI of 0.150, an entrapment efficiency of 75.6%, and a drug release of 93.04%. P-Ns-Opt’s vesicle morphology was discovered to be in the appropriate spherical form. The complete entrapment of plumbagin in niosomes was validated via thermal analysis data, and excipient compatibility was established based on the absence of chemical reaction among the excipients of niosomes and plumbagin. The improved formulation, known as P-Ns-Opt, showed intense release at first, but thereafter showed sluggish drug release in the in vitro drug release assessment. The Korsmeyer–Peppas, Higuchi, zero-order, and first-order models were among those examined. Additionally, P-Ns-Opt’s antioxidant activity was found to be 60.68 ± 0.02%, which was comparable to those of ascorbic acid (77.86 ± 0.03%) and plumbagin (49.59 ± 0.06). The in vitro antidiabetic activity (α-amylase) was analyzed, and P-Ns-Opt (90.69 ± 2.9%) demonstrated improved results as compared to plumbagin (83.64 ± 3.5%). Similarly, α-glucosidase assay was performed, and P-Ns-Opt displayed an 88.43 ± 0.89% inhibition percentage compared to plumbagin, i.e., 81.07 ± 1.2%, indicating that the developed formulation could effectively treat and manage diabetes.

The current study shows that the QbD technique may be used to manufacture plumbagin-loaded niosomes. BBD with a solvent evaporation procedure was applied to prepare and optimize the formulation. The expected and observed values were in agreement. The generated P-Ns-opts’ particle sizes will be suitable for oral administration. DSC and FTIR studies demonstrated that the produced P-Ns-opt contained plumbagin while preserving the formulation. The improved P-Ns-opt demonstrated good stability and sustained release. A dose-dependent inhibitory impact was also seen in antioxidant activity, in vitroα-amylase andα-glucosidase study. This study represents the first report of P-Ns-Opt formulation that investigated antioxidant and in vitro antidiabetic activity. In conclusion, it was determined that plumbagin-loaded niosomes are a more effective form of delivery of plumbagin and a better strategy for managing diabetes mellitus. Further studies using experimental animal models will facilitate the utilization of this formulation as a potent antidiabetic therapeutic agent.

## Figures and Tables

**Table 1:** Composition and optimization of P-Ns.

Formulation Codes	Independent Variables			Dependent Variables		
	Cholesterol (mg)	Span 20 (mg)	Sonication Time (min)	Vesicles Size (nm)	PDI	Entrapment Efficiency (%)
P-Ns-1	40	40	4	110.3	0.132	60.1
P-Ns-2	80	30	2	196.8	0.224	87.5
P-Ns-3	80	30	6	185.1	0.235	81.9
P-Ns-4	80	40	4	187.9	0.242	79.7
P-Ns-5	40	20	4	133.6	0.150	75.6
P-Ns-6	60	20	2	162.3	0.193	83.1
P-Ns-7	40	30	2	116.5	0.135	67.6
P-Ns-8	60	30	4	152.1	0.186	75.2
P-Ns-9	60	30	4	152.7	0.185	75.4
P-Ns-10	60	40	6	138.7	0.183	64.7
P-Ns-11	40	30	6	106.4	0.127	61.8
P-Ns-12	60	40	2	149.6	0.185	68.4
P-Ns-13	60	20	6	150.9	0.182	76.3
P-Ns-14	60	30	4	152.9	0.183	75.7
P-Ns-15	80	20	4	204.4	0.252	94.6

**Table 2:** Stability study of P-Ns-Opt at 4 ± 0.5 °C.

Observations	0 Days	30 Days	60 Days
Vesicle size	139.93 ± 6.77 nm	166.56 ± 6.35 nm	180.96 ± 1.89 nm
EE%	73.86 ± 4.1%	69.71 ± 1.5%	63.71 ± 2.7%

**Table 3:** Independent variables used to prepare and optimize P-Ns via Box–Behnken design.

Variables	Low	High
Independent variables		
X1 = Cholesterol (mg)	40	80
X2 = Span 20 (mg)	20	40
X3 = Sonication time (min)	2	6
Dependent variables		
Y1 = Vesicles size(nm)		
Y2 = PDI		
Y3 = Entrapment (%)		

## Data Availability

Data are contained within this article.
